# Supplementary material for: Inhibition of Polyamine Catabolism Reduces Cellular Senescence
Source: Int J Mol Sci. 2023 Aug 29;24(17):13397. doi: 10.3390/ijms241713397 (PMC10488189; doi:10.3390/ijms241713397)
Supplement: Supplementary file 1 [file ijms-24-13397-s001.zip › ijms-2565040-supplementary.pdf]

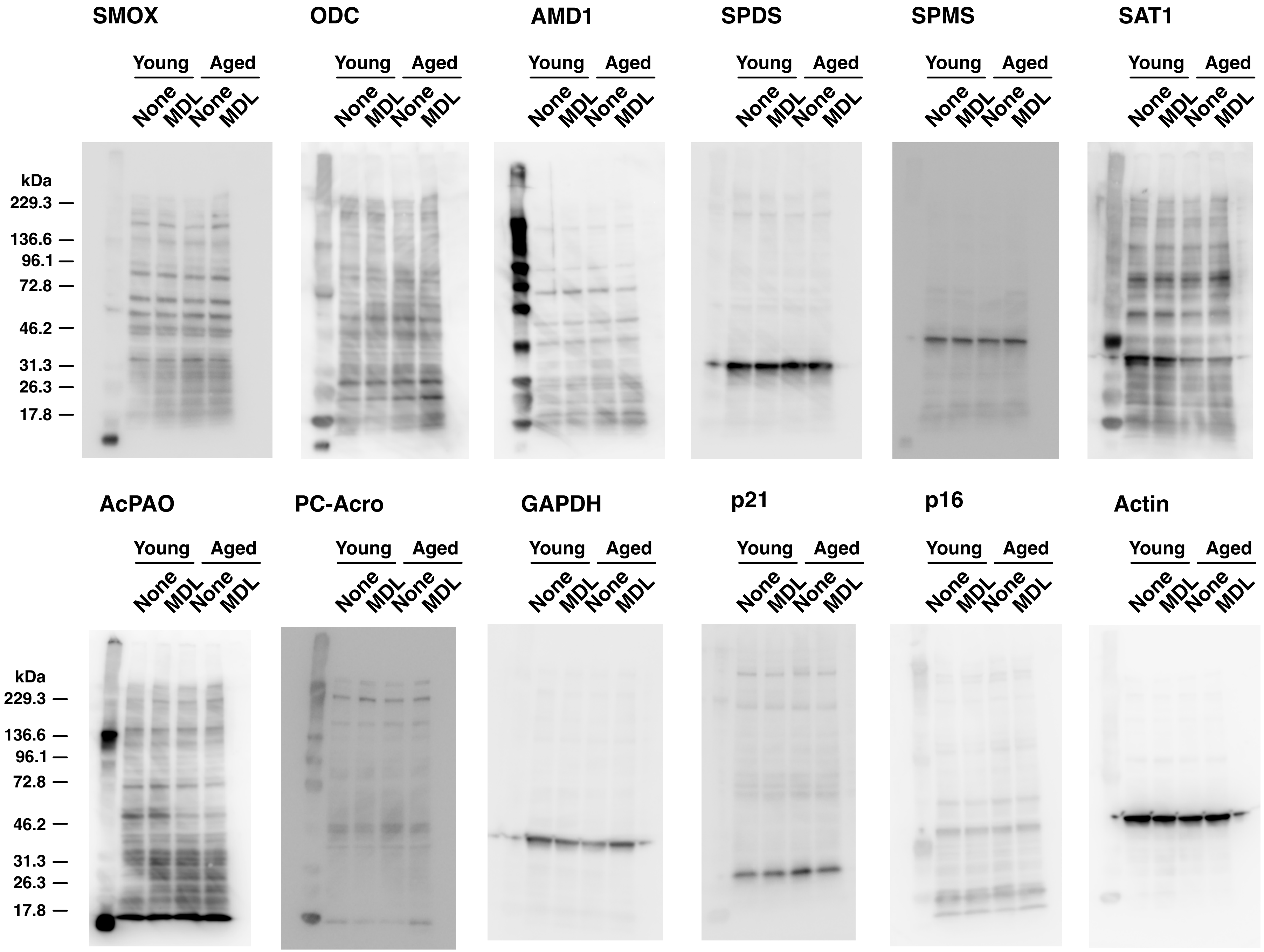

Supplemental Figure S1. The full length, non-adjusted images for SMOX, ODC, AMD1, SPDS, SPMS, SAT1, AcPAO, PC-Acro, GAPDH, p21, p16 and actin were shown.
